# Low values of central venous oxygen saturation (ScvO_2_) during surgery and anastomotic leak of abdominal trauma patients

**DOI:** 10.1186/s13017-017-0139-0

**Published:** 2017-06-19

**Authors:** Andres Isaza-Restrepo, Jose F. Moreno-Mejia, Juan S. Martin-Saavedra, Milciades Ibañez-Pinilla

**Affiliations:** 1 Escuela de Medicina y Ciencias de la Salud, Universidad del Rosario, Bogotá D.C., Colombia; 2 Mederi Hospital Universitario Mayor, Bogotá D.C., Colombia; 3 Hospital San Rafael, Tunja, Colombia; 4 Grupo de Investigación Clínica de la Universidad del Rosario, Bogotá, Colombia

**Keywords:** Abdominal injury, Perfusion, Hypotension, Anastomotic leak, Central venous pressure, Gastrointestinal tract

## Abstract

**Background:**

There is a well known relationship between hypoperfusion and postoperative complications like anastomotic leak. No studies have been done addressing this relationship in the context of abdominal trauma surgery. Central venous oxygen saturation is an important hypoperfusion marker of potential use in abdominal trauma surgery for identifying the risk of anastomotic leak development. The purpose of this study was to identify the relationship between low values of central venous oxygen saturation and anastomotic leak of gastrointestinal sutures in the postoperative period in abdominal trauma surgery.

**Methods:**

A cross-sectional prospective study was performed. Patients over 14 years old who required surgical gastrointestinal repair secondary to abdominal trauma were included. Anastomotic leak diagnosis was confirmed through clinical manifestations and diagnostic images or secondary surgery when needed. Central venous oxygen blood saturation was measured at the beginning of surgery through a central catheter. Demographic data, trauma mechanism, anatomic site of trauma, hemoglobin levels, abdominal trauma index, and comorbidities were assessed as secondary variables.

**Results:**

Patients who developed anastomotic leak showed lower mean central venous oxygen saturation levels (60.0% ± 2.94%) than those who did not (69.89% ± 7.21%) (*p* = 0.010).

**Conclusions:**

Central venous oxygen saturation <65% was associated with the development of gastrointestinal leak during postoperative time of patients who underwent surgery secondary to abdominal trauma.

## Background

Perioperative hemodynamic state, usually determined by mean blood pressure, urinary output, and heart rate, is highly related to postoperative (POP) morbidity and mortality [1–3], relationship that has been demonstrated in several studies [4–9]. Evidence has shown that an inadequate tissue perfusion during surgery is associated with anastomotic leak during the postoperative period, which is a major morbid complication [10]. In consequence, objective parameters of tissue perfusion are needed and central venous oxygen saturation (ScvO_2_) has been studied since the 1980 [4, 5].

Tissue hypoperfusion results from circulatory disturbances during hypoxic and hemorrhagic periods, and evidence suggests that ScvO_2_ reflects the early stages of hypoperfusion [6, 11–15]. Other hypoperfusion marker is lactic acid, which is elevated during low tissue oxygenation even under normal vital signs. Nevertheless, lactic acid could be elevated by different circumstances like hepatic impairment as well as those treated with Ringer lactate [15–18]. ScvO_2_, being an early hypoperfusion marker, could support early and objective decision-making during surgical procedure.

The main purpose of this study was to explore possible associations between tissue hypoperfusion identified through ScvO_2_ measurement and the development of anastomotic leak during the postoperative period of abdominal trauma patients. Our hypothesis is that low measurements (<65%) may increase the risk of presenting leakages of anastomosis or sutures during POP period.

## Methods

### Design, setting, and participants

Originally, the study was designed for a case-control study, but in response to the low incidence of cases during the study period, a prospective cross-sectional study approach was preferred. Patients aged 14 years or older, who underwent surgery secondary to abdominal trauma and that required primary gastrointestinal anastomosis, were included in the study. Patients were excluded when they presented hemodynamic or metabolic impairment secondary to previously known pathologies, and patients who died during surgery or in the immediate postsurgical time. As clinical and local conditions for gastrointestinal repair are different after damage control surgery, those that required this procedure before the final repair were also excluded. The study took place at the Hospital Occidente Kennedy, a reference trauma center of high complexity located in Bogotá D.C., Colombia, between 2011 and 2013. Patients underwent surgery according to each surgeon’s personal criteria and to the hospital’s trauma protocols without taking into account the study objectives.

### Variables

The following data was collected for every patient: age, gender, anatomic site of trauma, trauma mechanism, blood pressure, transfusion requirements, hemoglobin levels at arrival, comorbidities, abdominal trauma index (ATI), and the ScvO_2_ measured during the surgery. ScvO_2_ was measured through a subclavian venous catheter implanted by the anesthesiologist for central venous pressure measurement. As part of the hospital’s protocol, anesthesiologist prepared patients for surgery and implanted the subclavian venous catheter during the first 30 min of entering the surgery room. ScvO_2_ was measured right after catheter implantation. All remaining data was obtained from clinical records of every patient. Follow-up of patient was done during the hospitalization days until the first postoperative ambulatory control appointment 10 days after discharge or when anastomotic leak was diagnosed.

### Anastomotic diagnosis and ScvO_2_ cut point

Anastomotic leak or suture leakage was diagnosed from clinical manifestations and confirmed through image studies, or surgery during secondary interventions when needed. For this study’s main outcome, a ScvO_2_ cut point was defined as low levels previous to analysis. According to literature, normal levels range between 70 and 75% and levels under 60% suggest an oxidative metabolic disturbance [19]; therefore, the cut point for this study was set at 65%. All patients with ScvO_2_ at 65% or lower were classified as low levels.

### Sample selection and bias assessment

According to literature, incidence for anastomotic leak is ranged between 2.4 and 15.9% [1]. Therefore, considering a risk of 10% to develop anastomotic leak in abdominal trauma patients, a leaked/non-leaked relationship of 1:3; a type I error of 0.05 and type II of 0.010; and an overall final sample of 108 were calculated. Ultimately, due to low incidence of patients during the period of time for the study, an overall sample of 41 patients was reached and an approach for a cross-sectional prospective study was decided.

Selection bias was addressed through sequential and continuous selection of patients until a final sample of 41 patients was achieved taking into account inclusion and exclusion criteria. Hospital’s personnel that attended each case were not influenced by the objectives of the study and acted according to usual practice and protocols established in the hospital. Classification bias was addressed by taking into account data obtained from the clinical record of each patient.

### Statistical analysis

Qualitative variables were analyzed through descriptive statistics using absolute and relative frequencies. For quantitative variables, central tendency (means and median) and dispersion (rate and standard deviation) measurements were used. For assessing differences in systolic and diastolic arterial pressure, Student’s *t* test was used for two independent groups. Previous analysis of normalized values on arterial pressure and the homogeneity of variants were done using the Shapiro Wilk test and Levene test respectively. If such circumstances were not achieved, an exact non-parametric Mann Whitney test was used.

A Fisher exact test (expected values of <5) was applied for identifying any association between ScvO_2_ and the development of anastomotic leak. Risk was assessed using relative risk with a Katz exact confidence interval of 95%. Logistic regression and multivariate analysis was done, but due to the low number of patients, it was excluded from the reports.

## Results

### Participants and descriptive results

A total of 41 patients were analyzed and 4 of those (9.8%) developed an anastomotic leak. The mean age for the study was 32.83 ± 13.77 years (median = 28 years), 32 out of 41 (78%) were male and only 3 of the 41 (7.3%) had any comorbidity (see Table 1). Mean values for systolic blood pressure were 105 ± 14.4 mmHg and diastolic blood pressure were 67 ± 13 mmHg. Patients required red blood cell transfusion in 19.5% (*n* = 8) of the cases, and the mean values for hemoglobin at hospital arrival was 12.14 ± 2.77 (median of 13). Finally, the mean values for ScvO_2_ were 68.93 ± 7.5 (median of 70). Mean values for ATI was 5.34 ± 4.92 (median of 3) (see Table 2).

**Table 1:** Demographic data and red blood cell transfusion requirement

Anastomotic leak	Yes (*n* = 4)	No (*n* = 37)	*p* value
Gender male	2 (50%)	30 (81%)	0.204
Comorbidities	0 (0%)	3 (8.1%)	0.729
Mean age (SD)	34 (8.36)	32.7 (14.12)	0.503

No significant differences were observed

**Table 2:** Study outcomes

Anastomotic leak	Yes (*n* = 4)	No (*n* = 37)	*p* value
Hb (g/dL), mean (SD)	8.55 (2.59)	12.53 (2.52)	0.004*
ATI, mean (SD)	14.75 (5.62)	4.32 (3.66)	<0.001*
SBP (mmHg), mean (SD)	98.25 (16.64)	107.32 (14.20)	0.149
DBP (mmHg), mean (SD)	58.50 (16.66)	66.19 (12.66)	0.260
ScvO_2_ (%), mean (SD)	60.98 (2.94)	69.89 (7.21)	0.010*
Transfusion requirement, *N*	3 (75%)	5 (13.51%)	0.019*

*SD* standard deviation, *N* number of patients, *Hb* hemoglobin, *SBP* systolic blood pressure, *DBP* diastolic blood pressure, *ScvO*_*2*_ central venous oxygen saturation, *ATI* abdominal trauma index

*Significant difference

The most common site of trauma was the small intestine followed by the stomach and the colon (see Table 3). Main trauma mechanism was sharp weapon injury in 65.9% of the cases (*n* = 27), followed by firearm injury 22% (*n* = 9) and blunt trauma in 12.2% (*n* = 5) of the cases.

**Table 3:** Distribution of site of injury by organ

Organ	Frequency	Percent
Stomach	5	12.2
Small intestine	26	63.4
Ascending colon (including hepatic angle)	3	7.3
Tranverse colon (including splenic angle)	2	4.9
Descending colon	2	4.9
Sigmoid colon	3	7.3
Total	41	100

### Anastomotic leak and ScvO_2_

Out of the 41 patients, 7 (17.07%) evidenced surgical ScvO_2_ values <65% and 4 of those 7 patients presented anastomotic leak (see Table 4). Patients that presented anastomotic leak showed significantly lower (*p* = 0.010) values of ScvO_2_ during surgery (mean value of 60.0 ± 2.94%), compared to those with no anastomotic leak (mean value of 69.89 ± 7.21%). A significant association between values of ScvO_2_ <65% and the risk of developing an anastomotic leak was identified (RR = 39.8 CI 95%: 2.35, *p* < 0.0001, with a Fisher exact test).

**Table 4:** Distribution of cases according to ScvO_2_ saturation

		Anastomotic leak	
		Yes (total)	No (total)
Saturation	<65%	4 (7)	3 (7)
	≥65%	0 (34)	34 (34)
Total		4 (41)	37 (41)

### Other variables and ScvO_2_

No association was found with the presence of comorbidities (*p* = 0.729), gender (*p* = 0.204), age (*p* = 0.503), and systolic and diastolic blood pressure (*p* = 0.149 and *p* = 0.260). A significant association was identified with the requirement of red blood cell transfusion (*p* = 0.019), low values of hemoglobin at arrival (*p* = 0.004), and a greater ATI (*p* < 0.001).

## Discussion

An association between low levels of ScvO_2_ during surgery with anastomotic leak as a complication in the postsurgical period was found. Significant difference was identified between mean values of ScvO_2_ for patients that developed anastomotic leakages and those who did not (60.0% ± 2.94%, and 69.89% ± 7.21% respectively; *p* = 0.010). A risk of developing an anastomotic leak for patients with low levels of ScvO_2_ was identified (RR = 39.8 CI 95%: 2.35, *p* < 0.0001, with a Fisher exact test).

These results support the hypothesis that 65% or lower levels of ScvO_2_ may constitute a risk factor for the development of major complications related to gastrointestinal anastomosis and sutures in abdominal trauma patients. Other authors have found similar results [10, 11, 20–25]. Hoshking et al. showed that levels of 66.5% or lower of ScvO_2_ are associated with a worst outcome despite a stable hemodynamic state [15]. Pearse et al. described the changes in ScvO_2_ after mayor surgery finding an independent association between levels of ScvO_2_ (with a cut point of 65%) and the development of POP complications [11]. Other cases where low values of ScvO_2_ were associated with a worst prognosis are trauma, acute myocardial infarction [5, 26], severe sepsis, and cardiac failure [11].

In abdominal trauma, anastomotic leak is one of the most morbid complications [1, 27–30] and hypoperfusion is an important factor for its development. Consequently, different strategies have been developed for the prevention and resolution of hypoperfusion such as goal-directed resuscitation [9, 31] and goal-directed fluid replacement [25, 32].

We strongly believe that early and objective hypoperfusion markers can contribute to a supported decision on postponing primary repair. Based on the results from this study, ScvO_2_ monitoring during surgery could be a potential and easily available hypoperfusion marker. It is important to mention that values of ScvO_2_ may reflect a low oxygen extraction rate secondary to mitochondrial impairment indicating poor outcomes and limits low levels of ScvO_2_ sensibility [15, 33, 34].

Another limiting factor is the catheter placement. It has been proved that ScvO_2_ levels vary according to the catheter’s point location [35]; therefore, it is important to note that the sample was taken from the superior vena cava so measurements could be affected by brain metabolism, specially under anesthesia. Other potential marker is serum lactate, but it was not measured for this study as it can vary in different conditions such as fluid replacement with Ringer lactate [15–18], a common practice in trauma patients. Other limitations of this study were the sample size for the study and that ScvO_2_ values were not measured continuously.

A significant association with the requirement of red blood cell transfusion (*p* = 0.019), low values of hemoglobin at arrival (*p* = 0.004), and a greater ATI (*p* < 0.001) was also found. The first two variables are indirect markers of inadequate perfusion, further supporting its association with anastomotic leak development. This suggests that other markers may also help or may become cofounding factors for ScvO_2_ efficacy. Measurement of other markers (serum lactate, BE, CO_2_ gap, SaO_2_, and others), bigger samples, and a proper case-control or randomized controlled trials may be needed for answering these questions. Nonetheless, our results are robust enough to emphasize ScvO_2_ importance as a hypoperfusion marker and as a risk factor for POP anastomotic leakage in abdominal trauma surgery patients.

## Conclusions

Early recognition of hypoperfusion during abdominal trauma surgery is of great importance for adequate decision-making and for identification of patients at risk. This study shows that low ScvO_2_ levels (<65%) are associated with the development of anastomotic leak during the POP period and that ScvO_2_ may become an objective and accessible marker of hypoperfusion in patients with abdominal trauma. We consider further studies are required for a better understanding of the relationship between hypoperfusion and anastomotic leak, the use of ScvO_2_ as an early marker, and its usefulness for decision-making during surgery.

## Abbreviations

ATI: Abdominal trauma index
DBP: Diastolic blood pressure
Hb: Hemoglobin
POP: Postoperative
SBP: Systolic blood pressure
ScvO_2_: Central venous oxygen saturation
